# Comparative satisfaction and effectiveness of virtual simulation and usual supervised work for postpartum hemorrhage management: a crossover randomized controlled trial

**DOI:** 10.1186/s12909-022-03761-5

**Published:** 2022-10-06

**Authors:** Sandrine Voillequin, P. Rozenberg, K. Letutour, A. Rousseau

**Affiliations:** 1 Midwifery Department, CHRU Strasbourg, 67091 Strasbourg, France; 2 Department of Obstetrics and Gynecology, Poissy-Saint Germain Hospital, 78300 Poissy, France; 3 Clinical Epidemiology, Paris Saclay University, CESP, UVSQ, Inserm, Team U1018, 78180 Montigny- le-Bretonneux, France; 4 Midwifery Department, UVSQ, 78180 Montigny-le-Bretonneux, France

**Keywords:** Virtual simulation, Usual supervised work, Knowledge, Satisfaction

## Abstract

**Background:**

Because virtual simulation promotes learning and cognitive skill development, it may be useful for teaching students to manage postpartum hemorrhage (PPH) and its complex decision algorithm.

**Objective:**

This study aimed to compare the satisfaction and effectiveness of virtual simulation with usual supervised work in producing knowledge and satisfaction.

**Methods:**

This two-center two-stage crossover randomized controlled trial included student midwives. One group underwent the virtual simulation intervention in the first period (January 2018) and the usual supervised classroom work in the second (May 2018); the other group followed the reverse chronology. Satisfaction was the primary outcome. The secondary outcome was knowledge of the PPH management algorithm, assessed by responses to a case vignette after each intervention session.

**Results:**

The virtual simulation -supervised work (VS-SW) chronology was allocated to 48 students, and its inverse (SW-VS) to 47; Satisfaction was significantly higher for the virtual simulation for its overall grade (6.8 vs. 6.1, *P* = 0.009), engagingness (very good 82.1% vs. 24.3%, *P* < 0.001), and ease of use (very good 77.9% vs. 46.1%, *P* < 0.001). Knowledge did not differ between the two groups (respectively, 89.5% versus 83.5%, *P =* 0.3).

**Conclusion:**

Satisfaction is higher with virtual simulation without lowering knowledge scores, which argues for the use of such innovative teaching strategies. This could lead to an increase in students’ motivation to learn.

**Supplementary information:**

The online version contains supplementary material available at 10.1186/s12909-022-03761-5

## Background

Simulation methods are increasingly used in clinical teaching to immerse students in the delivery room before they encounter real-life situations [1]. Among the methods included in simulation pedagogy, virtual environments, serious games, and gamification are emerging innovative technologies that may offer multiple advantages over more traditional approaches [2–5]. Virtual simulation promotes learning and cognitive skill development in a virtual environment (visual and verbal) that gives meaning to what is learned, but is also engaging and easy to use. It allows learners to make decisions and acquire experience in a safe environment [2]. Virtual simulation may be especially interesting for situations with a complex decision algorithm requiring learners to choose between different options. It may also increase motivation and engagement [6].

Postpartum hemorrhage (PPH) is a frequent obstetric event, occurring in 5 to 10% of deliveries [7, 8]; when severe, it is a leading cause of maternal morbidity and mortality worldwide [9]. Reports from confidential inquiries have shown that 67% of PPH-related deaths in the United States and 84% of those in France were preventable; they resulted from delayed or inadequate management [10–12]. Despite similar international guidelines [13–15], variations in practices between and within countries have shown that efforts to improve the quality of maternity care are still needed [16–18]. Their effectiveness is particularly essential in initial training, especially as obstetrics remains one of the highest risk specialties in health care [19].

We thus wondered whether a virtual simulation could be a useful pedagogical tool to help student midwives in their initial professional training to learn how to manage PPH.

The aim of this study was to assess student satisfaction with virtual simulation compared to usual supervised work in learning PPH management. Our secondary objective was to compare the students’ knowledge needed to manage PPH and degree of certainty using clinical vignettes after each intervention.

## Methods

### Study design

We conducted a two-center, two-stage, crossover, randomized controlled trial. Student midwives were randomized into 2 groups. One group underwent the virtual simulation intervention in the first period (January 2019) and the usual supervised classroom work in the second (May 2019) while the other group underwent the usual supervised classroom work in the first period and the virtual simulation intervention in the second. We followed the CONSORT statement about randomized controlled trials in reporting this trial.

**Fig. 1:**
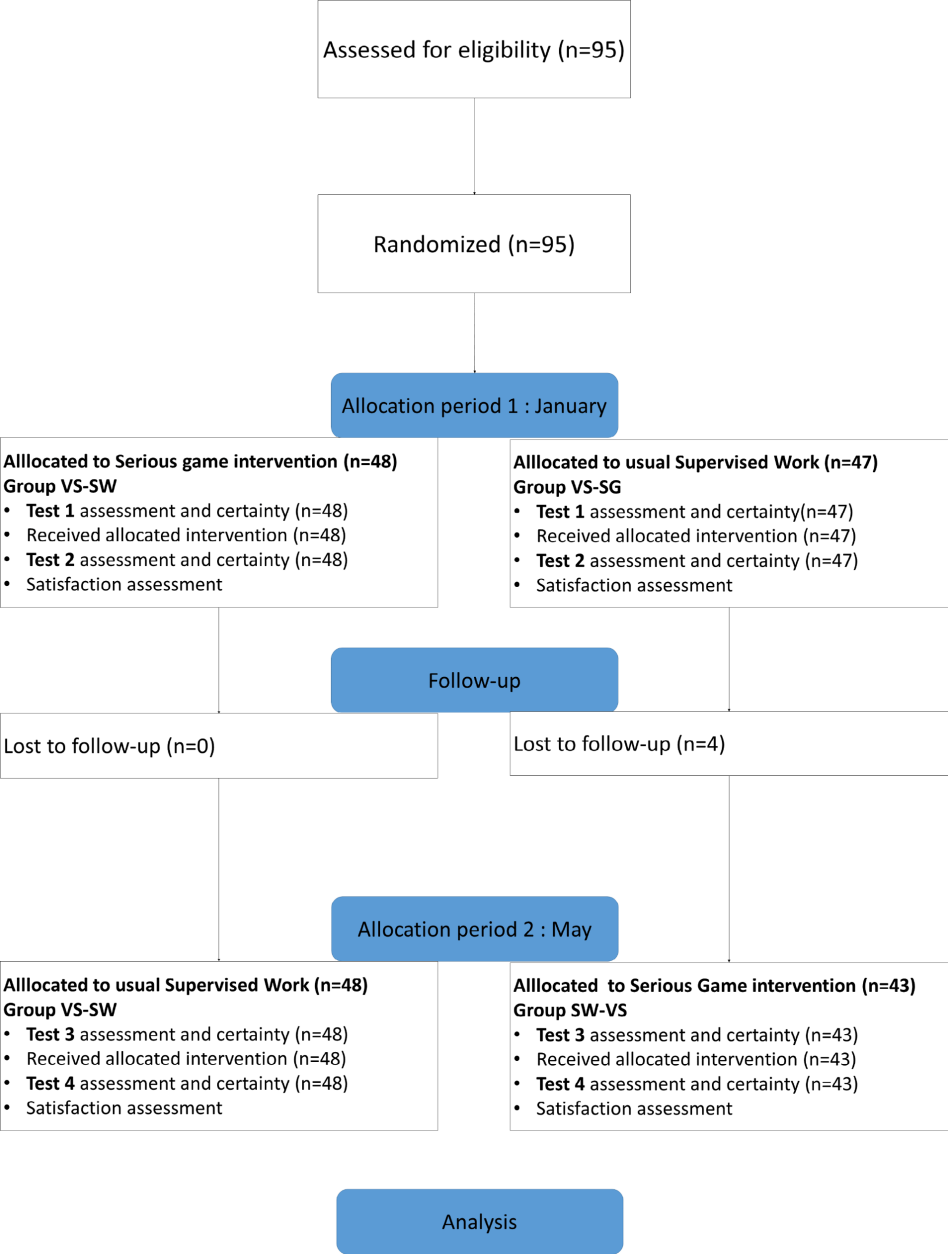
Consort flow diagram

In Fig. 1, the Consort flow diagram illustrates the participant flow during the different study stages. Flow for participants, withdrawals, and inclusion in analysis are described.

## Participants

The participants included in the study were student midwives in the 4th year of maieutic sciences from the Midwifery department at Versailles-Saint-Quentin University and the Midwives School in Strasbourg. The additional inclusion criterion was having received theoretical instruction in the form of courses; the exclusion criteria were prior exposure to this virtual simulation intervention or repeating the 4th year of study.

In France, midwives have 5 years of specific medical education, certified by a state diploma considered equivalent to a Master’s degree. They diagnose, provide initial management of PPH, and prescribe some medications at the same time as they call the obstetrician, with whom they work closely.

## Interventions

### Virtual simulation intervention

The Perinatsims virtual simulation (Medusims SAS)[20] offers users several PPH scenarios in the delivery room, in a 3-D and timed environment (see supplementary Files 1). It automatically generates a debriefing based on French best practice recommendations. During the simulation, the midwife is represented by an avatar who can move around the delivery room, perform various procedures, administer medications, call other professionals (obstetricians, anesthesiologists), and talk to the patient. The patient reacts to the different elements of treatment, and the situation deteriorates according to the management offered and the scenario underway.

The virtual simulation session was organized for 90 min in groups of 4–5 student midwives on a virtual simulator, playing out the same 2 scenarios of an immediate PPH for each group. With the first scenario, the students learned to handle the tool, and each group managed the second scenario autonomously. This session was followed by a debriefing focused on the elements of decision-making at the beginning of the PPH management (i.e., alert team members, administer oxytocin, manual placental delivery if necessary, uterine massage) and organization of care. The same midwife instructor conducted these games in each center (AR, SV).

The cost of the license for this virtual simulation is $1800 for classroom use.

### Usual supervised work

The same midwife in each center (AR, SV) supervised the standard classroom work, which also took 90 min and included half the usual full group of students (n = 15 or 16 rather than 30–32). In this specific session, based on a clinical situation, students were invited to discuss the case as part of analyzing the guidelines. Part of the discussion focused on the elements of decision-making at the beginning of the PPH management (i.e., alert team members, administer oxytocin, manual placental delivery, uterine massage) and organization of care. The classroom work did not contain hands-on components.

## Method of evaluation

The primary outcome was satisfaction. Satisfaction was measured by two self-administered questionnaires after each training stage (T2 and T4) with three items corresponding to: (1) global satisfaction, by a Likert scale (between 0 and 10); (2) its engagingness and appeal, and (3) its affordability and ease of use. These last two items were evaluated with multiple-choice questions with adjectives: very good, good, adequate, inadequate, with corresponding emojis. The 10-point Likert scale was chosen to offer a higher degree of measurement precision and provide a better opportunity to detect changes and more power to explain a point of view [21].

The secondary outcome was knowledge and degree of certainty of the PPH management algorithm, evaluated during the four assessment sessions by responses to a clinical case vignette, that is, a short, pragmatic clinical case that allows participants to immerse themselves in a situation as real as possible and be questioned about the management they would perform. This method has been previously validated and used to evaluate clinical practices in the context of PPH [18, 22]. Vignettes comprised a partogram describing the medical history, labor, delivery, and PPH and asked participants how they would manage the emergency situation with closed-ended question: “What actions would you perform within the next 15 minutes?” Then they have to choose none, one, or more actions from the list of choices for each type of management: pharmacological (antibiotic, oxytocin, misoprostol, sulprostone, or other), non-pharmacological (uterine massage, torsion of the cervix, bladder catheterization, manual examination of the uterine cavity, cervical examination with speculum, intrauterine tamponade, selective arterial embolization, surgical treatment), and communication, monitoring and investigation (alert other members of the team, venipuncture for blood sampling, resuscitation, and monitoring). (see supplementary Files 2 and 3)

The binary secondary outcome was evaluated by a composite outcome including 4 items expected in response to the clinical vignette: alert other members of the team, administer oxytocin, manually deliver the placenta and/or examine the uterine cavity, and massage the uterus. These 4 items were selected by expert consensus in a preliminary study [18] and correspond to important procedures that the midwife is responsible for performing.

These assessments took place before the beginning and after the end of each training stage. There were therefore 4 assessment sessions: T1 before the first training session, T2 after it, T3 before the second training session and T4 after it. Each test used a slightly different clinical PPH vignette but expected the same responses each time. To reduce the risk of contamination we administered out the test immediately after the training session and asked the participants not to communicate among themselves about the training sessions.

Degree of certainty was measured four times, at each assessment, by a multiple-choice question after the vignette: “how sure are you?“ with the following choices: <50%, 50–70%, 70–90%, > 90%.

## Sample size

We expected to see a 65% correct response rate to the supervised work (rate of appropriate oxytocin administration in the midwives’ survey [18]), and we were hoping for a higher rate after the virtual simulation (arbitrarily, 25% more), i.e., around 90%. If we assumed that the within-participant standard deviation of the response variable was 0.5 and that the probability that the study would detect a difference between the interventions was 80% at a two-sided 0.05 significance level, we would require a total of 90 students to participate in this two-intervention crossover study (45 in each arm).

## Randomization

Randomization took place after verification of the inclusion criteria and after participants had provided written informed consent. We used R software to draw up a randomization list by student class at each school. The distribution within the virtual simulation groups was random.

## Ethics

The National Data Protection Authority (‘Commission Nationale de l’Information et des Libertes’) approved this study on January 21, 2017 (CNIL number DN 17 − 01).

All study participants gave their written consent to take part. They were informed that they could withdraw their consent and/or their participation at any time.

### Statistical analysis

Categorical variables were described with numbers and percentages, and continuous variables by their means, standard deviations, and medians.

The two interventions (VS and SW) were compared for the main and secondary outcomes by pooling the evaluations conducted at T2 and T4. For dichotomous variables, chi-2 and Fisher exact tests were used as appropriate to assess differences in outcomes between groups. Student’s t and nonparametric tests were used to compare continuous outcomes between the two groups.

All statistical tests were two sided, and *P* < 0.05 was defined as statistically significant. Statistical analysis was conducted with R 3.6.2.

## Results

The virtual simulation-supervised work (VS-SW) chronology was allocated to 48 students, and its inverse (SW-VS) to 47. (Table 1) Four students in the second group were lost to follow-up. After the merger of T2 and T4 results for each method, we analyzed 95 students for the virtual simulation intervention and 91 for the usual supervised classroom work (Fig. 1).

**Table 1:** Students’ characteristics (N = 95)

		Group VS-SW	Group SW-VS	***P***-value
		N = 48	N = 47	
Age(years), mean (SD)		22.69 (1.8)	22.21 (0.7)	0.09
Age(years), median		2.22	2.22	
**Study location, n (%)**				
	UVSQ	32 (66.7)	32 (68.1)	NS
	Strasbourg	16 (33.3)	15 (31.9)	

The primary outcome of satisfaction was significantly higher for the virtual simulation training (Table 2), in particular for the global satisfaction grade (mean ± SD, 6.8 ± 1.6 vs. 6.1 ± 1.1 respectively, *P* = 0.009). These means explain why the score was dichotomized at 6.

At the end of the training (T2 + T4), the secondary outcome, composite knowledge, did not differ between the pooled virtual simulation group (n = 85/95, 89.5%) and the pooled usual supervised work group (n = 76/91, 83.5%, *P* = 0.3). The details of the results compared by test and according to the pedagogical tool are available in the appendix (supplementary file 4).

**Table 2:** Satisfaction (N = 95)

Variables		T2 + T4		
		Virtual simulation	Usual supervised work	***P***-value
		N = 95	N = 91	
		n (%)	n (%)	
**Satisfaction grade**				
	≤ 6	22 (23.2)	38 (45.8)^a^	0.002
	> 6	73 (76.8)	45 (54.2)^a^	
**Engagingness, appeal**				
	very good	78 (82.1)	23 (25.3)	< 0.001
	good	3 (3.2)	22 (24.2)	
	adequate	14 (14.7)	45 (49.4)	
	inadequate	0	1 (1.1)	
**Affordability, ease of use**				
	very good	74 (77.9)	42 (46.1)	< 0.001
	good	21 (22.1)	42 (46.2)	
	adequate	0	6 (6.6)	
	inadequate	0	1 (1.1)	

^a^ 4 missing data: 4 students did not respond to the satisfaction scale in test 4

**Fig. 2:**
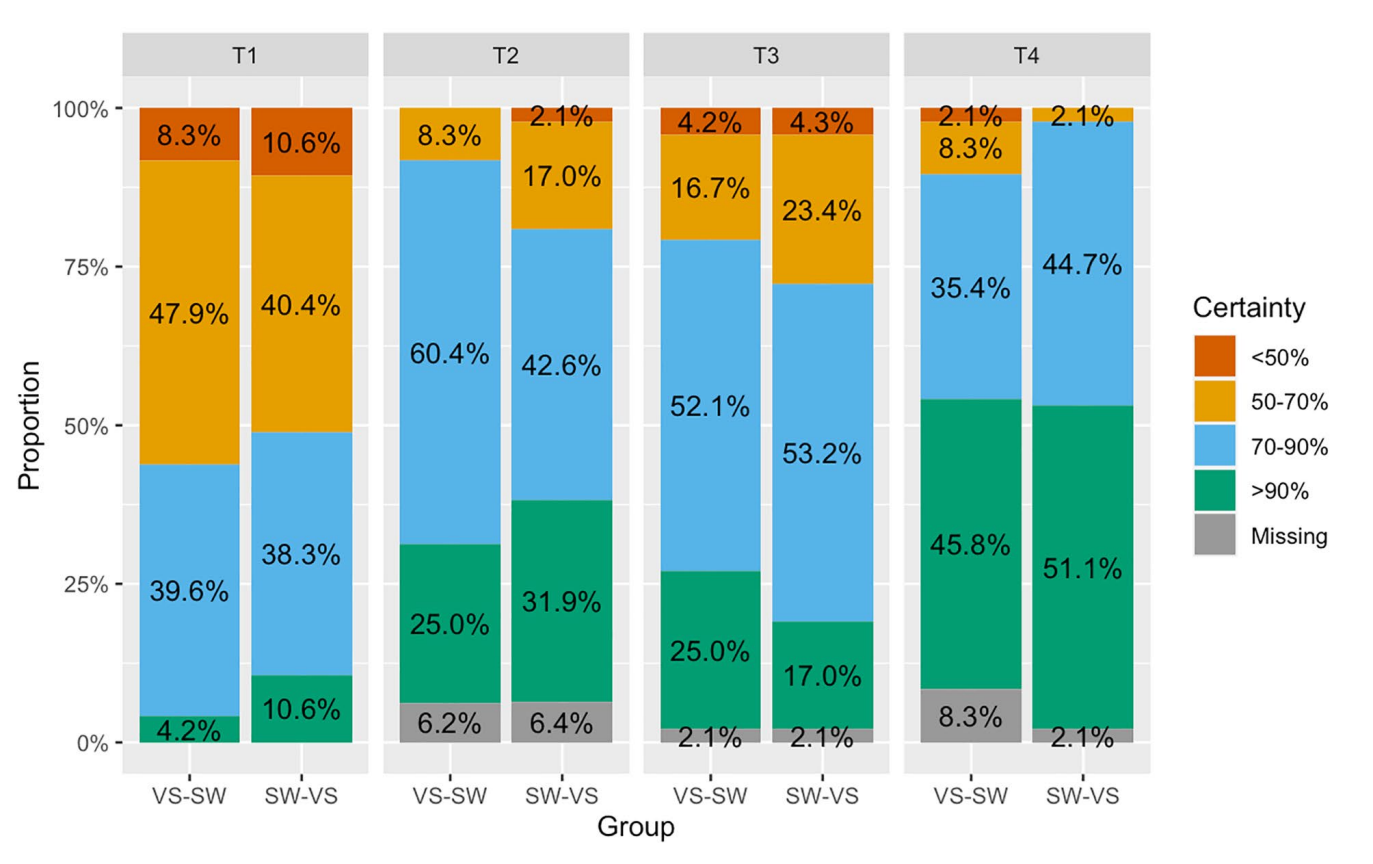
Degree of certainty reported by student midwives

Similarly, degree of certainty did not differ significantly between these two modes of instruction, with T2 + T4 pooled (*P* = 0.1), as shown in Fig. 2.

## Discussion

### Main findings

Students found the virtual simulation significantly more satisfying overall (grade of 6.8 ± 1.6 versus 6.1 ± 1.1, *P* = 0.009), engaging (very good 82.1% versus 25.3%, *P* < 0.001) and easy to use (very good 77.9% versus 46.1%, *P* < 0.001). Furthermore, knowledge of the algorithm for PPH management did not differ shortly after instruction by a virtual simulation or by the usual supervised work (*P* = 0.3).

Degree of certainty did not differ significantly after the two types of instruction, although Fig. 2 shows an improvement in certainty over time (T2 versus T4) and a slight decrease between the 2 teaching periods (over the 4 months between T2 and T3).

### Interpretation

The literature on the effects of virtual simulation remains mixed and of moderate methodological quality [23–25]. Nevertheless, several studies on their effectiveness have failed to show a difference in knowledge but did reveal higher satisfaction, in both initial and continuing medical education [5, 6, 26–31]. Gamification did not appear to enhance cognitive performance. The literature, like our study, shows that virtual simulation is more enjoyable and engaging, easier to use, and even has an effect on motivation. Students were more active during these simulations, immersed in a professional environment. Enjoyment is attractive to students and keeps them focused. This aspect was heightened in our study by the small size of the group (4–5) in the virtual simulation session, while the supervised work session included 15–16 students. The group may inhibit some students. Virtual simulation in small groups of students allow sociocentric learning, peer-to-peer.

Interactions between students and between students and the teacher also play an important role and may partly explain why knowledge levels did not differ between the virtual simulation and the supervised work. Moreover, both arms (VS and SW) used feedback and debriefing, helping to link the theoretical knowledge to its practical applications. Boeker et al.[32] showed that virtual simulation affected knowledge, but their script-based approach for their control group could not provide interactions. Virtual simulation can also be considered in team-based learning and even multiprofessional training. Team-based learning has proven effective in improving peer learning, teamwork, and communication skills [33, 34]. In our study, it is possible that the lack of difference in knowledge assessment between the virtual simulation and the supervised work is due to the close similarity of the two interventions, with only their formats different.

We observed a decrease in degree of certainty at time T3 (4 months after the first session). In their study of cardiac arrest, which included an assessment at 4 months, Drummond et al. [29] also suggested that some elements of management are only partially learned and retained. Several authors have shown that performance decreases 6 months after simulation training in learning resuscitation [35, 36]. Moreover, Raman et al. compared a dispersed (4 h spread out over four weeks) versus massed (one 4-hour half-day) course to promote better short and long-term retention [37]. They showed significantly better long-term knowledge through dispersed courses. These different results suggest that a regular use of virtual simulation would be useful for maintaining knowledge. The knowledge results at different times, especially at T3 (supplementary file 2 and 3 show the importance of repetition in knowledge retention. It seems that 2 trainings are not enough; an important advantage of virtual simulation is that students can perform it multiple times. Lack of time and resources prevent the repetition of supervised classroom work, but learners can replay virtual simulation independently, especially when they include integrated feedback, in both initial and continuing training, on the training site, the practice site or anywhere else. We could propose it as a distance learning alternative for students isolated due to their health condition or in cases of necessity, especially in situations such as the COVID-19 pandemic.

It has been shown that a “motivational dynamic model” increases engagement in training [38]. Motivation to learn is higher in students who see a benefit or usefulness in the activities they are asked to carry out (perception of the value of an educational activity). Virtual simulation seems to have a motivational potential due to the strong perception of engagingness. It should have positive effects on perceived self-efficacy, especially combined with other motivational strategies, such as encouraging success, promoting motivating assessments, and useful feedback.

### Strengths and limitations

Our study had several strengths. First, it was randomized. Next, in accordance with the definition by Djaouti et al. [39], the virtual simulation we used has an objective, rules, a result to achieve (that the woman bleed as little as possible and that all elements of the debriefing are correct, as shown by green font), and a playful design. In addition, our secondary outcome was based on a solid methodological tool of case-vignettes, previously validated in the specific context of postpartum hemorrhage [18, 22]. Finally it took place at two separate centers and was reasonably large.

Nonetheless, it also had some limitations. In particular, it would have been interesting to have had a third control group without any intervention to measure the changes between T2 and T3 in both groups. During this period, the students were in internships and might have had to deal with PPH situations or reviewed some earlier instruction after the intervention, which would have affected their knowledge on the subject. We did not have the necessary power to take into account this bias related to the internships and experiences between T2 and T3 because of the number of students. Nevertheless, the results presented in the Appendix show poorer results at T3 than at T2 and T1, thus demonstrating that this bias has a moderate impact and reinforcing the importance of learning by repetition. In addition, we used clinical vignettes to show differences in knowledge. Perhaps a simulation or some other actual practice-oriented testing method might have detected smaller differences. Lastly, students performed the virtual simulation in a small group, whereas the evaluation of the primary outcome was individual. We cannot know if individual sessions would have led to better learning with more knowledge, decision-making ability, and recall. Nevertheless, the collective competition was as present in the virtual simulation group as in the supervised work group.

## Conclusion

Satisfaction is higher with virtual simulation without lowering knowledge scores, which argues for the use of such innovative teaching strategies. Future studies should evaluate repeat use as self-stimulation alone or in teams to continue lifelong learning. Our results indicate that further studies should focus on analyzing the cost effectiveness of virtual reality teaching compared with traditional teaching.

## Practice points

- The virtual simulation is probably useful for learning how to manage postpartum hemorrhage because of its greater student satisfaction.
- The virtual simulation did not produce knowledge of postpartum hemorrhage management better than that obtained with the usual supervised work.
- Students’ degree of certainty increases over the course of learning but decreases between 2 learning sessions.

## Electronic supplementary material

Below is the link to the electronic supplementary material.

Supplementary Material 1

Supplementary Material 2

Supplementary Material 3

Supplementary Material 4

## Data Availability

The datasets used and analyzed during the current study are available from the corresponding author on request.

## **Abbreviations**

PPH: postpartum hemorrhage.
SW: supervised work.
VS: virtual simulation.
