# Supplementary figures and images for: Comparative satisfaction and effectiveness of virtual simulation and usual supervised work for postpartum hemorrhage management: a crossover randomized controlled trial
Source: BMC Med Educ. 2022 Oct 6;22:709. doi: 10.1186/s12909-022-03761-5 (PMC9540154; doi:10.1186/s12909-022-03761-5)

## Vignette 1

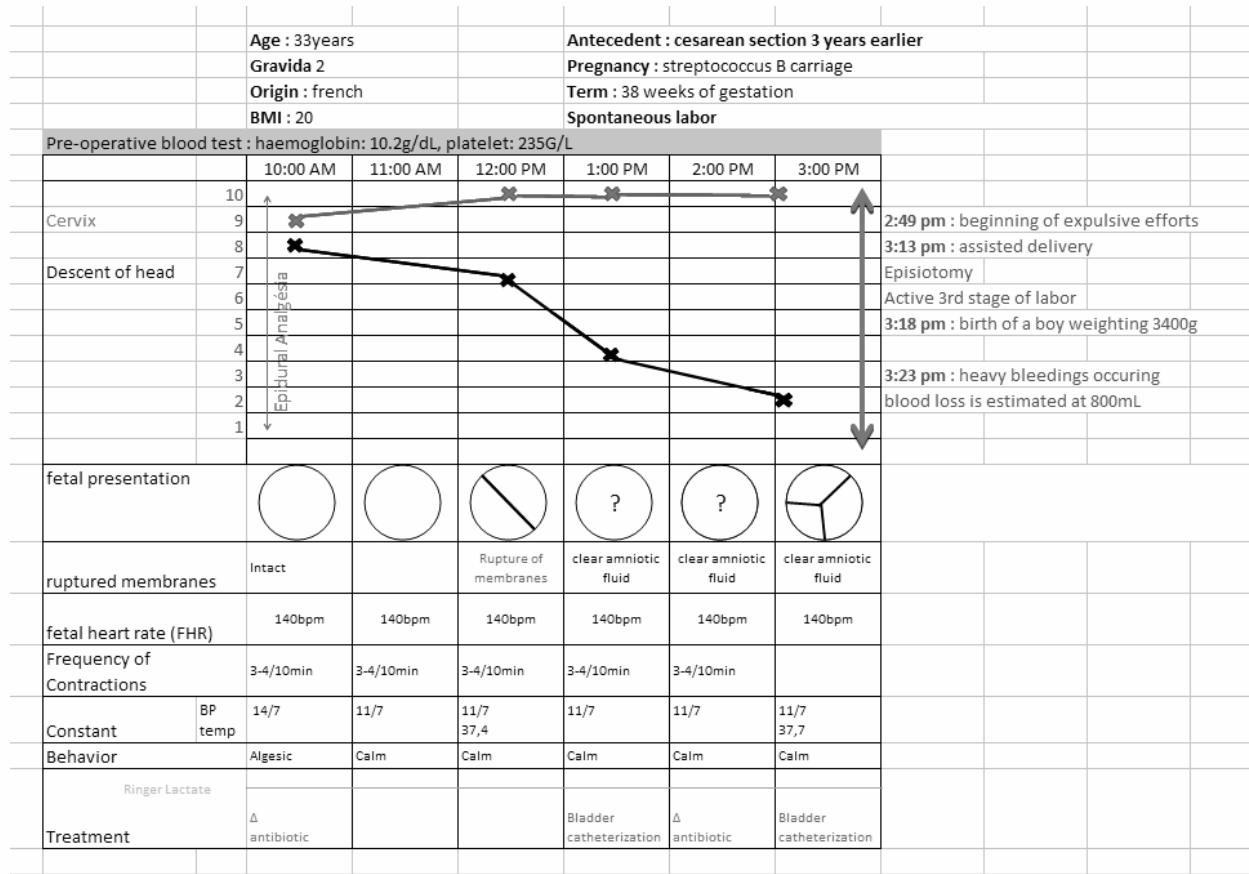

What measures would you perform **within the next 15 minutes** ?

Supplement: Supplementary file 1 — Supplementary Material 1 [file 12909_2022_3761_MOESM1_ESM.pdf]
