# Supplementary material for: Comparative satisfaction and effectiveness of virtual simulation and usual supervised work for postpartum hemorrhage management: a crossover randomized controlled trial
Source: BMC Med Educ. 2022 Oct 6;22:709. doi: 10.1186/s12909-022-03761-5 (PMC9540154; doi:10.1186/s12909-022-03761-5)
Supplement: Supplementary file 2 — Supplementary Material 2 [file 12909_2022_3761_MOESM2_ESM.pdf]

Vignette 2

Age : 31years
Gravida 1
Origin : Moroccan
BMI : 23

Course of pregnancy uneventful
Term : 40 weeks of gestation
Spontaneous labor

Pre-operative blood test : haemoglobin: 13.1g/dL, platelet: 178G/L

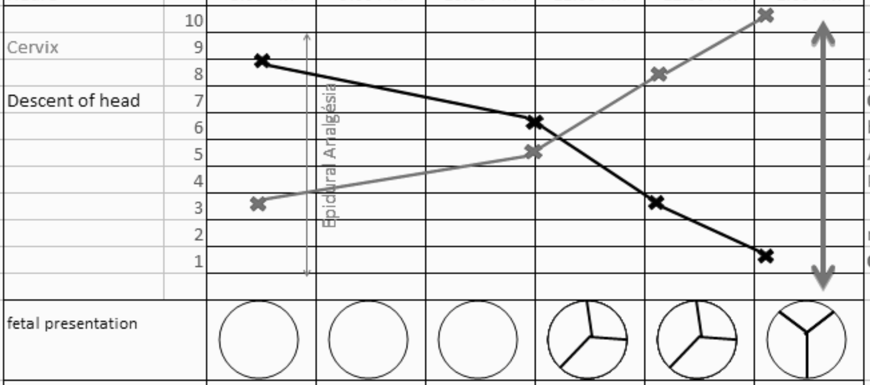

Heure	8:00 PM	9:00 PM	10:00 PM	11:00 PM	12:00 AM	1:00 AM
ruptured membranes		Rupture of membranes	clear amniotic fluid	clear amniotic fluid	clear amniotic fluid	brown amniotic fluid
fetal heart rate (FHR)	140bpm	140bpm	140bpm	140bpm	160bpm	160bpm
Frequency of Contractions	4/10min	3-4/10min	3-4/10min	3-4/10min	4/10min	4/10min
Constant BP	12/7	12/7	11/7	11/7	11/7	
Constant temp	36,9					
Behavior	Algic	Algic	Calm	Calm	Calm	Calm
Treatment (Ringer Lactate)				Oxytocine		Bladder catheterization

12:40 am: beginning of expulsive efforts
01:05 am : spontaneous vaginal delivery
boy weighting 3640g
Active 3rd stage of labor
Placenta delivered

minimal bleeding appears and stops with uterus massage
02:30 am : minimal bleeding persists, blood loss is estimated at 650mL

What measures would you perform **within the next 15 minutes** ?
