# Supplementary material for: Comparative satisfaction and effectiveness of virtual simulation and usual supervised work for postpartum hemorrhage management: a crossover randomized controlled trial
Source: BMC Med Educ. 2022 Oct 6;22:709. doi: 10.1186/s12909-022-03761-5 (PMC9540154; doi:10.1186/s12909-022-03761-5)

Supplementary file 1

Title : Screenshot of the virtual simulator

Legend : Patient lying in the delivery room with monitor screen


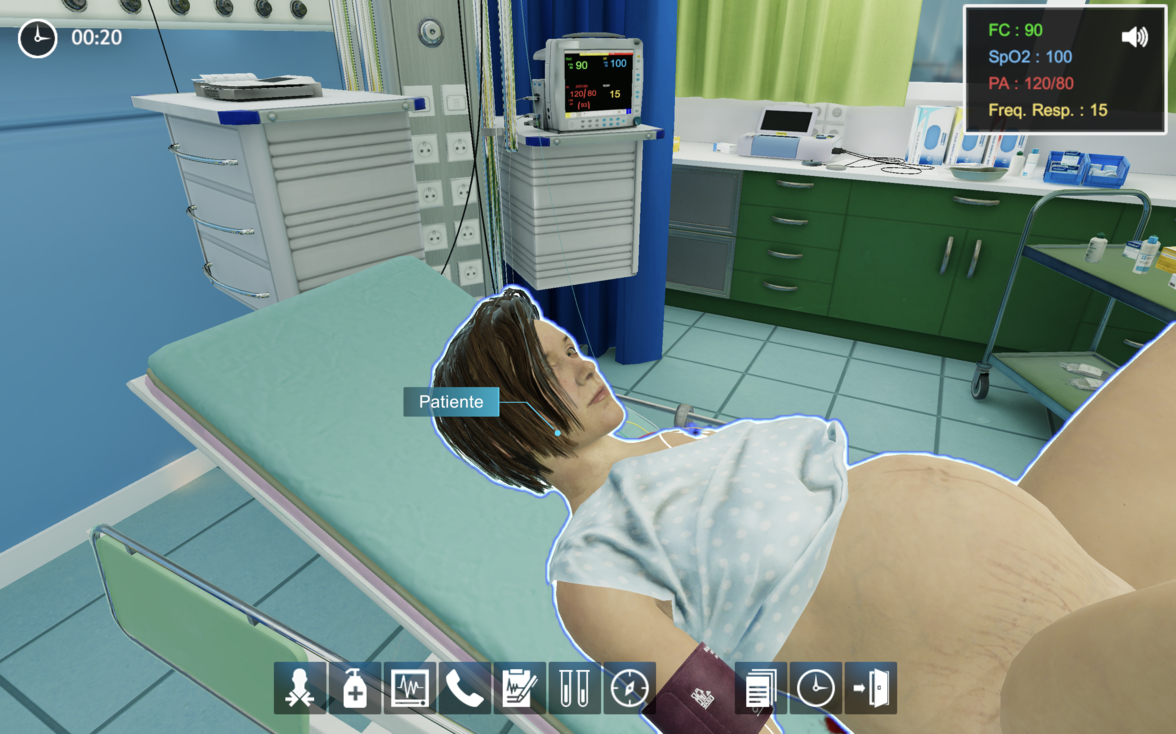

Supplement: Supplementary file 3 — Supplementary Material 3 [file 12909_2022_3761_MOESM3_ESM.docx]
