# Supplementary material for: Comparative satisfaction and effectiveness of virtual simulation and usual supervised work for postpartum hemorrhage management: a crossover randomized controlled trial
Source: BMC Med Educ. 2022 Oct 6;22:709. doi: 10.1186/s12909-022-03761-5 (PMC9540154; doi:10.1186/s12909-022-03761-5)
Supplement: Supplementary file 4 — Supplementary Material 4 [file 12909_2022_3761_MOESM4_ESM.docx]

Supplementary Files 4

Title : Adherence to guidelines for each test

|  | Adherence to guidelines | | | | | |
| --- | --- | --- | --- | --- | --- | --- |
|  | T1 | T2 | *P*-value | T3 | T4 | *P*-value |
|  | n(%) | n(%) |  | n(%) | n(%) |  |
| Virtual Simulation (N=47) | 37(77) | 44(92) | 0.08 | 26(55) | 41(87) | <.01 |
| Usual supervised work (N=48) | 38(81) | 38(81) | >.9 | 25(52) | 38(86) | <.01 |

Legend : Comparison between T1 and T3 and then T3 and T4 for each method

Title : Adherence to guidelines for each method

|  | Adherence to guidelines | | |
| --- | --- | --- | --- |
|  | Virtual Simulation | Usual supervised work | *P*-value |
|  | N=47 | N=48 |  |
|  | n(%) | n(%) |  |
| Tests |  |  |  |
| T1 | 37(77) | 38(81) | 0.7 |
| T2 | 44(92) | 38(81) | 0.13 |
| T3 | 25(52) | 26(55) | 0.8 |
| T4 | 41(87) | 38(86) | >.9 |

Legend : Comparison between each method for each test
